# Semiautomated TaqMan PCR screening of GMO labelled samples for (unauthorised) GMOs

**DOI:** 10.1007/s00216-017-0333-7

**Published:** 2017-04-17

**Authors:** Ingrid M. J. Scholtens, Bonnie Molenaar, Richard A. van Hoof, Stephanie Zaaijer, Theo W. Prins, Esther J. Kok

**Affiliations:** RIKILT Wageningen University & Research, P.O. box 230, 6700 AE Wageningen, The Netherlands

**Keywords:** Screening, Specificity, Element, Construct, GMO, Automation

## Abstract

In most countries, systems are in place to analyse food products for the potential presence of genetically modified organisms (GMOs), to enforce labelling requirements and to screen for the potential presence of unauthorised GMOs. With the growing number of GMOs on the world market, a larger diversity of methods is required for informative analyses. In this paper, the specificity of an extended screening set consisting of 32 screening methods to identify different crop species (endogenous genes) and GMO elements was verified against 59 different GMO reference materials. In addition, a cost- and time-efficient strategy for DNA isolation, screening and identification is presented. A module for semiautomated analysis of the screening results and planning of subsequent event-specific tests for identification has been developed. The Excel-based module contains information on the experimentally verified specificity of the element methods and of the EU authorisation status of the GMO events. If a detected GMO element cannot be explained by any of the events as identified in the same sample, this may indicate the presence of an unknown unauthorised GMO that may not yet have been assessed for its safety for humans, animals or the environment.

**Electronic supplementary material:**

The online version of this article (doi:10.1007/s00216-017-0333-7) contains supplementary material, which is available to authorized users.

## Introduction

An increasing number of genetically modified organisms (GMOs), e.g. soy, maize, canola, potato, are currently grown worldwide, with many others in the process of development [1]. In the European Union (EU), strict labelling requirements are in place. Authorised GMOs are allowed up to a level of 0.9% in non-GMO material, provided their presence is unintentional [2]. There is a zero tolerance level for unauthorised GMOs in food products. In feed some GMOs that are not (yet) authorised but are in the process of authorisation are allowed up to a level of 0.1% [3]. This status can be given if there is already an authorisation in another country, if positive advice with relation to the safety of the particular GMO is given by the European Food Safety Authority (EFSA), and if reference materials and a method for identification of the GMO are available.

For enforcement of the labelling requirements practical detection and quantification methods for routine laboratories are needed. Several novel DNA-based techniques, including micro-arrays, digital PCR and next generation sequencing (NGS), are described for GMO detection and identification [4–10]. Nevertheless quantitative polymerase chain reaction (qPCR) is still the most widely used technique for GMO screening, identification and quantification in routine GMO laboratories. The availability of interlaboratory validated event-specific TaqMan PCR methods for all EU authorised GMOs is an important reason for this. For every GMO event that is authorised in the EU, an event-specific TaqMan qPCR method that targets the sequence bridging the GMO insert and the plant DNA and thus identifying one specific GMO is obligatorily provided by the manufacturer of the GMO in question. These methods were verified by the European Union Reference Laboratory for Genetically Modified Food and Feed (EURL-GMFF) and underwent interlaboratory validation by the EURL-GMFF with the aid of the European Network of GMO laboratories (ENGL) prior to their use for official control (http://gmo-crl.jrc.ec.europa.eu/StatusOfDossiers.aspx). Also for GMOs that are in the process of being authorised, EURL interlaboratory validated methods are available in several cases.

The GMO insert typically consists of a combination of a number of genetic elements derived from several species (promoter, regulator, coding sequence and terminator(s) from bacteria, viruses, plants) to obtain new characteristics in the plant, e.g. herbicide or insect resistance. The series of joint elements is called the genetic construct, and construct-specific methods identify two (or more) joint elements of a particular construct that may be present in different GMOs. Because testing on all possible GMO events is time consuming and impractical, laboratories first screen for elements and constructs that are commonly used, applying well-established qPCR methods. This strategy is called the matrix approach [11–14]. Several screening strategies for GMOs, using qPCR with TaqMan probes or SYBR Green I, have been described [15–20] and also pre-spotted screening plates have been developed on the basis of this strategy [21, 22]. For cost-efficient screening of non-GMO labelled samples for the presence of authorised and unauthorised GMOs, for which event-specific methods are available, a screening approach using a minimal amount of GM elements and constructs that detects all known GMOs may be used [12]. However, using this approach it is unlikely that indications for unknown unauthorised GMOs will be obtained. With only a few frequently used GMO elements, chances are considerable that the presence of unauthorised GMOs may be masked by the presence of (multiple) GMOs in the same sample. As a result, for the screening of GMO labelled samples that will contain GMOs, this minimal screening step is not informative enough to obtain indications for the presence of (unknown) unauthorised GMOs. For screening of whole series of GMO labelled samples this minimal screening step is not informative enough since the samples are bound to contain GMO elements. For GMO labelled samples an extended screening with many elements and constructs potentially reduces the number of subsequent event-specific tests needed. At the same time, when not all elements can be explained by known GMO events as identified in the same sample this gives an indication of the presence of an unknown unauthorised GMO in the sample.

An extended screening strategy for whole series samples, utilizing 32 screening PCRs, is presented in this paper. For GMO labelled samples, when all elements and constructs are explained by the known authorised and unauthorised events detected in the sample, no further events need to be tested, thus reducing the workload per sample. If an element cannot be explained by any of the known events identified in the sample, this is an indication of the potential presence of an unknown unauthorised GMO. A cost- and time-effective semiautomated strategy to screen and analyse a series of 11 samples using a DNA extraction robot, PCR plate pipetting robot and a semiautomated Microsoft Excel module for result analyses is presented here. The screening results of 11 samples and 32 tests can be copied into the Excel module. The Excel module predicts the potentially present events on the basis of detected screening elements. After the identified events are entered in the module it indicates if all elements are explained and summarizes the results of all tests performed on the 11 samples in a table. This table can be used to send all results to a laboratory information management system (LIMS).

## Materials and methods

### Reference materials for specificity verification

The Certified Reference Materials (CRM) were obtained from the Institute for Reference Materials and Measurements (IRMM, Geel, Belgium), the American Oil Chemists’ Society (AOCS, Urbana, Illinois, USA) and Fluka (Sigma-Aldrich, Saint Louis, MI, USA). Positive material of the unauthorised DAS59132 (E32) maize was obtained from the EURL-GMFF (Ispra VA, Italy). The CRMs used are described in Table 1. The reference materials are intended for detection and quantification of the specified GMO event and not for specificity testing. Low level contamination of these materials with other GMO events cannot be excluded.

**Table 1:** Reference materials used for specificity verification of element and construct screening methods

Supplier code	% GMO	GMO event
AOCS 0707-B	>99.99% soy leaf DNA	A2704-12 soy
AOCS 0707-C	>99.99% soy leaf DNA	A5547-127 soy
AOCS 0911-C	96.32% soy powder	CV127 soy
ERM-BF436b	98.6% soy powder	DAS44406 soy
ERM-BF432d	10% soy powder	DAS68416 soy
ERM-BF437b	98.6% soy powder	DAS81419 soy
ERM-BF426d	10% soy powder	DP305423 soy
ERM-BF425d	10% soy powder	DP356043 soy
AOCS 0610-A	>99.99% soy leaf DNA	FG72 soy
ERM-BF410g	10% soy powder	GTS 40-3-2 soy
AOCS 0809-A	>99.94% soy powder	MON87701 soy
AOCS 0210-A	>99.4% soy powder	MON87705 soy
AOCS 0311-A	>99.05% soy powder	MON87708 soy
AOCS 0809-B	>99.94% soy powder	MON87769 soy
AOCS 0906-B	>99.40% soy powder	MON89788 soy
ERM-BF420c	9.8% maize powder	3272 maize
AOCS 0411-D	>99.88% maize powder	5307 maize
ERM-BF412f	4.89% maize powder	Bt11 maize
ERM-BF411f	5% maize powder	Bt176 maize
Fluka 69407	1% maize DNA	CBH351 maize
ERM-BF433d	10% maize powder	DAS40278 maize
ERM-BF424d	9.78% maize powder	DAS59122 maize
JRC	1% maize DNA	DAS59132 (E32) maize
ERM-BF427d	10% maize powder	DP98140 maize
ERM-BF414f	4.29% maize powder	GA21 maize
AOCS 1208-A	>99.88% maize powder	MIR162 maize
ERM-BF423d	9.85% maize powder	MIR604 maize
ERM-BF413f	5% maize powder	MON810 maize
ERM-BF416d	9.85% maize powder	MON863 maize
AOCS 0512-A	>99.94% maize powder	MON87427 maize
AOCS 0709-A	>99.05% maize powder	MON87460 maize
AOCS 0406-D	>99.05% maize powder	MON88017 maize
AOCS 0906-E	>99.425% maize powder	MON89034 maize
ERM-BF415f	4.91% maize powder	NK603 maize
AOCS 0306-H	>99.99% maize powder	T25 maize
ERM-BF418d	9.86% maize powder	TC1507 maize
ERM-BF434b	98.8% canola powder	DP73496 canola
AOCS 1011-A	>99.94% canola powder	MON88302 canola
AOCS 0711-A	>99.99% canola leaf DNA	Ms1 canola
AOCS 0306-F	>99.99% canola leaf DNA	Ms8 canola
AOCS 0711-B	>99.99% canola leaf DNA	Rf1 canola
AOCS 0711-C	>99.99% canola leaf DNA	Rf2 canola
AOCS 0306-G	>99.99% canola leaf DNA	Rf3 canola
AOCS 0304-B	>99.19% canola powder	GT73 canola
AOCS 0208-A	>99.99% canola leaf DNA	T45 canola
AOCS 0711-D	>99.99% canola leaf DNA	TOPAS 19/2 canola
ERM-BF421b	100% potato powder	EH92-527-1 potato
ERM-BF422d	10% cotton powder	281-24-236 × 3006-210-23 cotton
ERM-BF428c	10% cotton powder	GHB119 cotton
AOCS 1108-A	>99.99% cotton leaf DNA	GHB614 cotton
AOCS 0306-E	>99.99% cotton leaf DNA	LL25 cotton
AOCS 0804-B	>99.4% cotton powder	MON1445 cotton
AOCS 0804-D	>98.45% cotton powder	MON15985 cotton
AOCS 0804-C	>97.39% cotton powder	MON531 cotton
AOCS 0906-D	>99.4% cotton powder	MON88913 cotton
ERM-BF429c	10% cotton powder	T304-40 cotton
EURL-GMFF	0.1% rice DNA	LL601 rice
AOCS 0306-I	>99.99% rice leaf DNA	LL62 rice
ERM-BF419b	100% sugar beet powder	H7-1 sugar beet

### DNA isolation

Several CRMs were obtained as leaf DNA and no further treatment was carried out. From CRMs that were obtained as powder, DNA was isolated from 100 ± 10 mg dry material using the DNeasy Plant Mini Kit (Qiagen, Venlo, Netherlands) according to the manufacturer’s protocol. For crops other than soy the lysis step with the manufacturer’s AP1 buffer was replaced with cetyl trimethylammonium bromide (CTAB) extraction buffer (20 g/L CTAB, 1.4 M NaCl, 0.1 M Tris, 20 mM Na_2_EDTA, pH 8.0). Incubation time of the CTAB extraction buffer is 30 min at 65 °C, and after 15 min of incubation 20 μL of 20 mg/mL proteinase K was added. Alternatively, DNA from soy, maize, canola and cotton was isolated from 100 ± 10 mg dry material using the Maxwell® 16 MDx instrument (Promega, Madison WI, USA) with the custom-made Maxwell® 16 Food Feed Seed (FFS) Nucleic Acid Extraction System according to the manufacturer’s protocol (now available as Maxwell® RSC PureFood GMO and Authentication Kit). The lysis step was performed with 1 mL CTAB extraction buffer, 40 μL Proteinase K 20 mg/mL (FFS kit) and 20 μL RNase A solution 4 mg/mL (Qiagen, 100 mg/mL). The incubation time is 90 min at 65 °C. After this step the cartridge from the FFS kit is loaded and fed into the Maxwell instrument according to manufacturer’s protocol. Extracted DNA concentrations and quality were measured on a NanoDrop spectrophotometer (Thermo Scientific, Waltham, MA, USA). The concentration was further diluted to 10 ng/μL in water (ultrapure distilled water, DNase and RNase free, Life Technologies, USA) and stored at 4 °C or −20 °C. DNA quality was checked using the *A*_260_/*A*_280_ and *A*_260_/*A*_230_ ratios. Although the Qiagen kit can be used for feed samples the Maxwell DNA extraction system is proposed for series of feed samples, because using the DNA extraction robot saves hands-on time. Feed samples include processed and unprocessed soy, maize, canola products and mixed feed.

### Primers and probes

The primers and probe sequences of all methods used in the routine screening strategy for GMO labelled feed samples are shown in Table 2. Primers and probes were ordered from Biolegio (Nijmegen, Netherlands) or Eurogentec (Belgium). All probes were labelled at the 5’ end with 6-carboxyfluorescein (FAM) dye and at the 3’ end with 6-carboxytetramethylrhodamine (TAMRA) quencher. The final concentrations of the primers and probes were as described in the literature or adjusted to 400 nM for both primers and 200 nM for the probes (Table 2). Using the QIAgility robot it was feasible to use optimal conditions for each method, but for several (older) methods 400 nM for both primers and 200 nM for the probes were used [23].

**Table 2:** Primer and probe sequences of the methods used in routine GMO feed samples screening strategy

Method	Primers and probe	Sequence 5’–3’	Final conc. (nM)	Reference
Plant actin	Act-f	CAAGCAGCATGAAGATCAAGGT	400	[27]
	Act-r	CACATCTGTTGGAAAGTGCTGAG	400	
	Act-probe	FAM-CCTCCAATCCAGACACTGTACTTYCTCTC-TAMRA	200	
Canola FatA**	FatA primer1	GGTCTCTCAGCAAGTGGGTGAT	400	[28]
	FatA primer2	TCGTCCCGAACTTCATCTGTAA	400	
	FatA probe	FAM-ATGAACCAAGACACAAGGCGGCTTCA-TAMRA	200	
Maize HMG	ZM1-F	TTGGACTAGAAATCTCGTGCTGA	400	[28]
	ZM1-R	GCTACATAGGGAGCCTTGTCCT	400	
	Probe ZM	FAM-CAATCCACACAAACGCACGCGTA-TAMRA	200	
Rice SPS	SPS-f	TTGCGCCTGAACGGATAT	400	[29]
	SPS-r	CGGTTGATCTTTTCGGGATG	400	
	SPS-P2	FAM-TCCGAGCCGTCCGTGCGTC-TAMRA*	200	
Soy Lec	Lec F	CCAGCTTCGCCGCTTCCTTC	400	[28]
	Lec R	GAAGGCAAGCCCATCTGCAAGCC	400	
	Lec P	FAM-CTTCACCTTCTATGCCCCTGACAC-TAMRA	200	
Sugar beet GS	GluA3-F	GACCTCCATATTACTGAAAGGAAG	400	[28]
	GluA3-R	GAGTAATTGCTCCATCCTGTTCA	400	
	GluD1 probe	FAM-CTACGAAGTTTAAAGTATGTGCCGCTC-TAMRA	200	
Wheat Wx-1	wx012-5'	GTCGCGGGAACAGAGGTGT	400	[30]
	wx012-3'	GGTGTTCCTCCATTGCGAAA	400	
	wx012-T	FAM-CAAGGCGGCCGAAATAAGTTGCC-TAMRA	200	
P-35S	P35S-1-5’	ATTGATGTGATATCTCCACTGACGT	400	[26]
	P35S-1-3’	CCTCTCCAAATGAAATGAACTTCCT	400	
	P35S-Taq	FAM-CCCACTATCCTTCGCAAGACCCTTCCT-TAMRA	200	
P-FMV	P-FMV-F	CGAAGACTTAAAGTTAGTGGGCATCT	340	[17]
	P-FMV-R	TTTTGTCTGGTCCCCACAA	340	
	P-FMV-P	FAM-TGAAAGTAATCTTGTCAACATCGAGCAGCTGG-TAMRA	540	
P-nos	P-NOS-F	GTGACCTTAGGCGACTTTTGAAC	340	[17]
	P-NOS-R	CGCGGGTTTCTGGAGTTTAA	340	
	P-NOS-P	FAM-CGCAATAATGGTTTCTGACGTATGTGCTTAGC-TAMRA	540	
P-Rice actin	P-Rice actin-F	TCGAGGTCATTCATATGCTTGAG	340	[17]
	P-Rice actin-R	TTTTAACTGATGTTTTCACTTTTGACC	340	
	P-Rice actin-P	FAM-AGAGAGTCGGGATAGTCCAAAATAAAACAAAGGTA-TAMRA	540	
P-SSuAra	P-SSuAra-F	GGCCTAAGGAGAGGTGTTGAGA	340	[17]
	P-SSuAra-R	CTCATAGATAACGATAAGATTCATGGAATT	340	
	P-SSuAra-P	FAM-CCTTATCGGCTTGAACCGCTGGAATAA-TAMRA	540	
T-CaMV 35S	T-35S-F	AGGGTTTCTTATATGCTCAACACATG	340	[17]
	T-35S-R	TCACCAGTCTCTCTCTACAAATCTATCAC	340	
	T-35S-P	FAM-AAACCCTATAAGAACCCTAATTCCCTTATCTGGGA-TAMRA	540	
T-E9	T-E9-F	TGAGAATGAACAAAAGGACCATATCA	340	[17]
	T-E9-R	TTTTTATTCGGTTTTCGCTATCG	340	
	T-E9-P	FAM-TCATTAACTCTTCTCCATCCATTTCCATTTCACAGT-TAMRA	540	
T-g7 (=T-ORF1)	T-g7-F	ATGCAAGTTTAAATTCAGAAATATTTCAA	340	[17]
	T-g7-R	ATGTATTACACATAATATCGCACTCAGTCT	340	
	T-g7-P	FAM-ACTGATTATATCAGCTGGTACATTGCCGTAGATGA-TAMRA	540	
T-nos	NOS ter 2-5’	GTCTTGCGATGATTATCATATAATTTCTG	400	[26]
	NOS ter 2-3’	CGCTATATTTTGTTTTCTATCGCGT	400	
	NOS-Taq	FAM-AGATGGGTTTTTATGATTAGAGTCCCGCAA-TAMRA	200	
Cp4-epsps	epsps 1-5'	GCCTCGTGTCGGAAAACCCT	400	[23]
	epsps 3-3'	TTCGTATCGGAGAGTTCGATCTTC	400	
	epsps-probe	FAM-TGCCACGATGATCGCCACGAGCTTCC-TAMRA	200	
Cry1A(b)	cry1A 4-5'	GGACAACAACCCMAACATCAAC	400	[23]
	cry1A 4-3'	GCACGAACTCGCTSAGCAG	400	
	Cry1A(b)-probe	FAM-CATCCCGTACAACTGCCTCAGCAACCCTG-TAMRA	200	
Cry1a.105	Cry1a.105-F1	TCAGAGGTCCAGGGTTTACAGG	400	[18]
	Cry1A.105-R1	GTAGTAGAGGCATAGCGGATTCTTG	400	
	Cry1A.105	FAM-AGACATTCTTCGTCGCACAAGTGGAGGACC-TAMRA	200	
Cry1Ab/Ac	Bt-F1	GAGGAAATGCGTATTCAATTCAAC	400	[31]
	Bt-R	TTCTGGACTGCGAACAATGG	400	
	Bt-P	FAM-ACATGAACAGCGCCTTGACCACAGC-TAMRA	200	
Cry1F	Cry1F-F2	GACGTGGATCTTCATCTGCAATC	400	[23]
	Cry1Fr-n2	GCAACACGGCTGGCAATCG	400	
	Cry1F-P2	FAM-CGCCCCCGGGATTGAAGACCCCGTAAC-TAMRA	200	
Cry2Ab2	Cry2Ab2-F	AATTCTAACTACTTCCCCGACTACTTC	400	[18]
	Cry2Ab2-R	ACGGAGAGGCGATGTTCCTG	400	
	Cry2Ab2-P	FAM-TCTCTGGTGTTCCTCTCGTCGTCCGCA-TAMRA	200	
Cry3Bb1	Cry3Bbf-n2	CCGCCCAGGACTCCATCG	400	[23]
	Cry3Bbr-n2	GAGGCACCCGAGGACAGG	400	
	Cry3BbP-n3	FAM-CTGCCGCCTGAGACCACTGACGAGC-TAMRA	200	
Vip3A	Vip3A-F2	TCACCAAGAAGATGAAGAC	400	[8]
	Vip3A-R2	CTCTCCACCTTCTTCTTG	400	
	Vip3A-P	FAM-TGACCGCCAACTTCTACGACA-TAMRA	200	
Bar	bar 2-5'	ACTGGGCTCCACGCTCTACA	400	[32]
	bar 2-3'	AAACCCACGTCATGCCAGTTC	400	
	Bar-1-Taq	FAM-ATGCTGCGGGCGGCCGGCTTCAAGCACGG-TAMRA	200	
Pat	Patf-n2	GACAGAGCCACAAACACCACAA	400	[5]
	Patr-n2	CAATCGTAAGCGTTCCTAGCCT	400	
	Patp-n2	FAM-GCCACAACACCCTCAACCTCA-TAMRA	200	
NptII	npt 1-5'	GACAGGTCGGTCTTGACAAAAAG	400	[23]
	npt 1-3'	GAACAAGATGGATTGCACGC	400	
	nptII-probe	FAM-TGCCCAGTCATAGCCGAATAGCCTCTCCA-TAMRA	200	
I-rAct1	AINT 2-5'	TCGTCAGGCTTAGATGTGCTAGA	400	[32]
	AINT 2-3'	CTGCATTTGTCACAAATCATGAA	400	
	AINT-2-Taq	FAM-TTTGTGGGTAGAATTTGAATCCCTCAGC-TAMRA	200	
Barstar	BstarF-n2	AACAAATCAGAAGTATCAGCGACCT	400	[5]
	BstarR-n2	AACTGCCTCCATTCCAAAACG	400	
	BStarP-n3	FAM-ACCTGGACGCTTTATGGGATT-TAMRA	200	
CaMV	CaMV-F1	TGAAATCCTCAGTGACCAAAAATC	300	[33]
	CaMV-R1	TACAAGGACAATCATTGATGAGC	300	
	CaMV-pr1	FAM-AAGCCGTTGCAGCGAAAATCGTTAATGA-TAMRA	200	
Ctp2/CP4-epsps	GT73-TmF	GGGATGACGTTAATTGGCTCTG	375	[34]
	GT73-TmR	GGCTGCTTGCACCGTGAAG	375	
	GT73-TmP	FAM-CACGCCGTGGAAACAGAAGACATGACC-TAMRA	150	
Ctp4/CP4-epsps	RRS 01-5’	CCTTTAGGATTTCAGCATCAGTGG	500	[26]
	RRS 01-3’	GACTTGTCGCCGGGAATG	500	
	RRS-Taq	FAM-CGCAACCGCCCGCAAATCC-TAMRA	200	

*Reverse complement probe compared to Ding et al. [29] as described in ISO21579:2005/Amd.1:2013(E) [35].

**Document taken from [28], but now available on http://www.monsanto.com/products/documents/dna-detection/canola_dna_dm.pdf

**Table 4:** Specificity of screening methods determined with available reference materials (Table 1)

Crop	Element/construct																									GMO event
	P-35S	P-FMV	P-nos	P-Rice actin	P-SSuAra	T-35S	T-E9	Tg7 (T-ORF1)	T-nos	CP4-epsps	Cry1A(b)	Cry1A.105	Cry1Ab/Ac	Cry1F	Cry2Ab2	Cry3Bb1	Vip3a	bar	pat	nptII	I-rAct1	Barstar	CaMV	ctp2/CP4-epsps	ctp4/CP4-epsps	
Soy	+	–	–	–	–	+	–	–	–	–	–	–	–	–	–	–		–	+	–	–	–	–	–		A2704-12 soy
	+	–	–	–	–	+	–	–	–	–	–	–	–	–	–	–		–	+	–	–	–	–	–		A5547-127 soy
	–	–	–	–	–	–	–	–	–	–	–	–	–	–	–	–			–	–		–	–	–		CV127 soy*
	–	–	–	–	–	–	–	+	–	–	–	–	–	–	–	–	–	–	+	–	–	–	–	–		DAS44406 soy
						–		+		–	–	–	–	–	–	–		–	+	–			–	–		DAS68416 soy
						–		+		–	–	–	**X**	**X**	–	–		–	+	–			–	–		DAS81419 soy
	–	–	–	–	–	–	–	–	–	–	–	–	–	–	–	–		–	–	–	–	–	–	–		DP305423 soy*
	**X**	–	–	–	–	–	–	–	–	–	–	–	–	–	–	–		–	–	–	–	–	–	–		DP356043 soy*
		–	–	–	–	–	–	–	+	–	–	–	–	–	–	–	–	–	–	–	–		–	–		FG72 soy
	+	–	–	–	–	–	–	–	+	+	–	–		–	–	–		–	–	–	–	–	–	–	+	GTS 40-3-2 soy
	–	–	–	–	+	–	–	–	–	–	–	–	+	–	–	–	–	–	–	–	–	–	–	–	–	MON87701 soy
	–	+	–	–	–	–	+	–	–	**X**	–	–	–	–	–	–	–	–	–	–	–	–	–	+	–	MON87705 soy
	–		–	–	–	–	+	–	–		–	–	–	–	–	–			–	–		–	–	–	–	MON87708 soy
	–		–	–	–	–	+	–	–	–	–	–	–	–	–	–	–	–	–	–	–	–	–	–		MON87769 soy
	–	+	–	–	–	–	+	–	–	**X**	–	–	–	–	–	–		–	–	–	–	–	–	+	–	MON89788 soy
Maize	–	–	–	–	–	**X**			+	–	–	–	–	–	–	–	–	–	–	–	–	–	–	–		3272 maize
										–	**X**	–	+	–	–	–		–	–	–			–	–		5307 maize
	+	–	–	–	–	+*	–	–	+	–	**X**	–	+	–	–	–		–	+	–	–	–	–	–		Bt11 maize
	+	–	–	–	–	**X**	–	–	–	–	+	–	**X**	–	–	–	–	+	–	–	–	–	–	–		Bt176 maize
	+					(+)			+	–		–		–	–	–		+	–	–	–	–	–			CBH351 maize
								–		–	–	–	–	–	–	–		–	–	–			–	–		DAS40278 maize*
	+	–	–	–	–	+	–	–	–	–	–	–	–	–	–	–		–	+	–	–	–	–	–		DAS59122 maize
	+					(+)			–	–	–	–	–	–	–	–		–	+	–	–	–	–			DAS59132 (E32) maize
	–	–	–	–	–	–	–	–	–	–	–	–	–	–	–	–		–	–	–	–	–	–	–		DP98140 maize*
	–	–	–	+	–	–	–	–	+	–	–	–	–	–	–	–		–	–	–	+		–	–		GA21 maize
	–	–	–	–	–	**X**	–	–	+	–	–	–	–	–	–	–	+	–	–	–	–	–	–	–		MIR162 maize
	–	–	–	–	–	–	–	–	+	–	–	–	–	–	–	–		–	–	–	–	–	–	–		MIR604 maize
	+	–	–	–	–	–	–	–	–	–	+		**X**	–	–	–	–	–	–	–	–	–	–	–		MON810 maize
	+	–	–	–	–	–	–	–	+	–	–	–	–	–	–	+	–	–	–	–	+		–	–		MON863 maize
	+			–		–		–	+	**X**	–	–	–	–	–	–		–	–	–			–	+		MON87427 maize
	+		–	**X**		–	–		+	–	–	–	–	–	–	–		–		+	+			–		MON87460 maize
	+	–	–	+	–	–	–	–	+	+	–	–	–	–	–	+	–	–	–	–	+	–	–	+	–	MON88017 maize
	+	+	–		–	–	–	–	+	–	+	+	**X**		+	–	–	–	–	–	+	–	–	–		MON89034 maize
	+	–	–	+	–	–	–	–	+	+	–	–	–	–	–	–	–	–	–	–	+	–	–	+		NK603 maize
	+	–	–	–	–	+	–	–	–	–	–	–	–	–	–	–		–	+	+	–	–	–	–		T25 maize
	+	–	–	–	–	+	–	–	–	–	–	–	–	+	–	–	–	–	+	–	–	–	–	–		TC1507 maize
Canola		–	–	–	–	–	–	–		–	–	–	–	–	–	–			–					–		DP073496 canola*
	–	+	–			–	+	–	–	**X**	–	–	–	–	–	–		–	–	–			–	+	–	MON88302 canola
	–	–	+	–	+	–	–	+	+	–	–	–	–	–	–	–	–	+	–	+	–		–	–		Ms1 canola
	–	–	–	–	+	–	–	+	+	–	–	–	–	–	–	–		+	–	–	–		–	–		Ms8 canola
	–	–	+	–	+	–	–	+	+	–	–	–	–	–	–	–	–	+	–	+	–	+	–	–		Rf1 canola
	–	–	+	–	+	–	–	+	+	–	–	–	–	–	–	–	–	+	–	+	–	+	–	–		Rf2 canola
	–	–	–	–	+	–	–	+	+	–	–	–	–	–	–	–		+	–	–	–	+	–	–		Rf3 canola
	–	+	–	–	–	–	+	–	–	**X**	–	–	–	–	–	–		–	–	–	–	–	–	+	–	GT73 canola
	+	–	–	–	–	+	–	–	–	–	–	–	–	–	–	–		–	+	–	–	–	–	–		T45 canola
	+	–	+	–	–	+	–	–		–	–	–	–	–	–	–			+					–		TOPAS 19/2 canola
Potato	–	–	+	–	–	–	–	–	+	–	–	–	–	–	–	–		–	–		–	–	–			EH92-527-1 potato
	–	–	–	–	–	–	–	–	–	–	–	–	**X**	+	–	–	–	–	+	–	–	–	–	–		281-24-236x3006-210-23 cotton**
	+	–	–	–	–	+	–	–	+	–	–	–	–	–	–	–		+		–						GHB119 cotton
		–	–	–	–	–	–	–		–	–	–	–	–	–	–	–	–	–	–						GHB614 cotton*
	+	–	–	–	–	–	–	–	+	–	–	–	–	–	–	–	–	+	–	–	–	–	–	–	–	LL25 cotton
	+	+	–	–	–	–	+	–	+	**X**	–	–	–	–	–	–	–	–	–	+	–	–	–	+	–	MON1445 cotton
	+		–	–	–	–	–	–	+	–	–	–	+	–	+	–	–	–	–	+	–	–	–	–		MON15985 cotton
	+		–	–	–	–	–	–	+	–	–	–	+	–	–	–	–	–	–	+	–	–	–	–		MON531 cotton
	+	+	–	–	–	–	+	–	–	**X**	–	–		–		–		–	–					+	–	MON88913 cotton
Cotton	+	–	–	–	–	–	–	–	+		+	–	–	–	–	–		+		–						T304-40 cotton
Rice				(+)					+		+		+								(+)					Bt63 rice construct
	+			(+)		+			–	–	–	–	–	–	–	–		+	–	–	(+)	–	–			LL601 rice
	+	–	–	+	–	+	–	–	–	–	–	–	–	–	–	–		+	–	–	+	–	–			LL62 rice
Sugar beet	–	+	–	–	–	–	+	–	–	**X**	–	–	–	–	–	–		–	–	–	–	–	–	+		H7-1 sugar beet

*Italic: no screening elements, event testing necessary

**: combined because only stack reference material available; 281-24-236 contains cry1F, pat; 3006-210-23 contains cry1Ac, pat

+, detected; –, not detected; **X**, contains the element but it is not detected because of sequence differences; +*, detected because targets may have sequence similarities; empty, expected negative but not verified experimentally; (+), expected positive but not verified experimentally because of lack of reference material

### Limits of detection

The screening methods should be able to detect less than 0.045% or 25 copies [24]. For the published methods the limits of detection (LOD) were verified according to the ENGL method verification document [25] using 10-fold repetitions close to the expected LOD and found to be fewer than 20 copies (in-house validation data not shown). The LODs of methods designed and published by RIKILT Wageningen University & Research were not verified again because they were already validated in our laboratory on the basis of 60 repetitions at LOD level [8, 18]. To monitor the sensitivity of the methods in the sample analysis a positive sensitivity control for every screening method was used in each screening series at the level of 0.1% GMO or 25 copies (0.1% GMO CRM at 50 ng per PCR reaction, or higher percentage GMO diluted to 25 haploid genome equivalent copies per reaction in non-GMO background DNA). For practical reasons a control of 50 ng DNA isolated from 0.1% IRMM material was used where possible (=44 copies soy (1C = 1.13 pg), 18 copies maize (1C = 2.725 pg), 43 copies canola (1C = 1.15 pg). The actual number of copies in the 0.1% reference material depends on the zygosity.

**Fig. 1:**
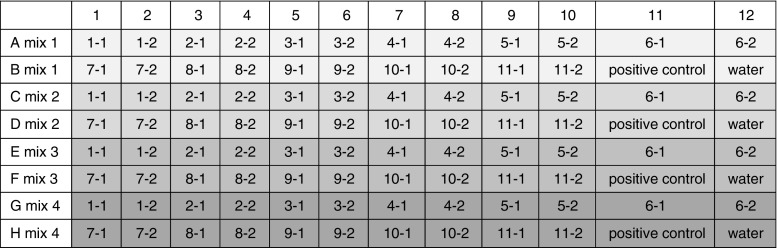
Uniform setup for eight 96-well PCR plates to screen 11 samples in duplicate (sample numbers indicated as 1-1 to 11-2), including positive sensitivity controls (0.1% or 25 copies) and negative controls (water), on four screening methods per plate

The 32 positive sensitivity controls, one well for each screening method, were added to the eight screening plates with four different screening methods (see plate setup in Fig. 1). The positive control for a screening method was chosen from one of the certified GMO reference materials that contained this element. Some 0.1% GMO positive controls were used as control for several methods. The following CRMs were used as positive control for the screening methods at a level of 50 ng 0.1%, if available as CRM, or at 25 copies in a background of 50 ng non-GMO material: MON810 maize for *Cry1A*(*b*); MON863 maize for *Cry3Bb*1, *npt*II, I-*rAct*1; NK603 maize for P-Rice actin, T-*nos*, ctp2/CP4-*epsps*; MON810 maize for P-35S, CP4-*epsps*, ctp4/CP4-*epsps*; TC1507 maize for T-35S, Cry1F, pat; CaMV positive sample for CaMV; MON87701 soy for P-*SSuAra*, Cry1Ab/Ac; MON89034 maize for Cry1A.105, *Cry2Ab*2; MON89788 soy for P-FMV(2), T-E9; Rf1 canola for P-*nos*, T-g7 (T-ORF1), bar, barstar; MIR162 for Vip3a; common wheat for endogenous gene wheat *Wx*-*1*; non-modified canola (AOCS0306B) for endogenous gene canola *FatA*; DP305423 for endogenous gene soy *Lec*; H7-1 for endogenous gene sugar beet *GS*; MIR162 for endogenous gene maize *hmg*; non-modified rice (AOCS0306D) for endogenous gene rice *SPS*; A2704 for the general plant *actin*.

### QIAgility robot

For pipetting the screening plates the QIAgility robot (Qiagen) was used with Qiagen QIAgility software version 4.17.1. The 32 screening methods were pipetted in eight 96-well plates (Table 3). Every run of the QIAgility prepares four PCR mixes to test four screening methods on 11 samples and a positive and negative control. The QIAgility robot is loaded with Mastermix (1 × 1.5-mL Eppendorf tube), water (1 × 5-mL tube), primers and probes for four screening methods (12 × 1.5-mL Eppendorf tube), four empty tubes to make the mixes (4 × 1.5-mL Eppendorf tube), four positive controls (4 × 1.5-mL Eppendorf tube), 11 samples in two isolations (22 × 1.5-mL Eppendorf tube), 1 × 200-μL conductive filtered tips (Qiagen, cat. #990522), 2 × 50-μL conductive filtered tips (Qiagen, cat. #990512) and an empty 96-well plate (Bio-Rad cat #HSP-9645). First the four mixes for four screening methods consisting of Mastermix, water, two primers and a probe were prepared and were divided over the 96-well plate in 4 × 24 wells by the QIAgility robot (Fig. 1). Then the DNA from two different DNA isolations per sample of 11 samples and the positive sensitivity controls (0.1% GMO or 25 copies) and a water control were pipetted by the QIAgility robot (Fig. 1).

**Table 3:** Distribution of screening methods over eight 96-well PCR plates

Plate	Mix	Method	plate	Mix	Method
1	1	Plant actin	5	1	Cry1A(b)
1	2	Canola FatA	5	2	Cry1A.105
1	3	Maize HMG	5	3	Cry1Ab/Ac
1	4	Rice SPS	5	4	Cry1F
2	1	Soy Lectin	6	1	Cry2Ab2
2	2	Sugar beet GS	6	2	Cry3Bb1
2	3	Wheat Wx-1	6	3	Vip3A
2	4	P-35S	6	4	Bar
3	1	P-FMV	7	1	Pat
3	2	P-nos	7	2	NptII
3	3	P-SSuAra	7	3	I-rAct1
3	4	T-CaMV 35S	7	4	Barstar
4	1	T-E9	8	1	CaMV
4	2	T-g7 (T-ORF1)	8	2	Ctp2/CP4-epsps
4	3	T-Nos	8	3	Ctp4/CP4-epsps
4	4	CP4-epsps	8	4	P-Rice actin

### Mastermixes

The QIAgility robot pipets a total reaction volume of 25 μL in every well consisting of 20 μL of the mix and 5 μL of the DNA (10 ng/μL). The mixes were prepared with 2× Diagenode Mastermix (Real time PCR Master Mix, cat. #DMMM-2X-A300, Seraing, Belgium), and the primers (10 μM) and probes (10 μM) were added to the final concentrations as mentioned in Table 2 and further diluted in water (ultrapure distilled water, DNase and RNase free, Life Technologies, USA) to a final volume of 20 μL per well. After the DNA was added, the 96-well plate is covered with a Bio-Rad seal (Microseal® 'B' Adhesive Seals cat. #MSB1001), vortexed briefly and centrifuged at 1000 rpm for 1 min to remove air bubbles.

### PCR

Real-time PCR reactions were performed on Bio-Rad CFX 96 machines (Bio-Rad Laboratories Inc, Hercules, CA, USA) with Bio-Rad CFX Manager 3.1 software. The PCR program consisted of a decontamination step with UNG for 120 s at 50 °C, activation of the Taq polymerase for 600 s at 95 °C, followed by 45 cycles of 15 s at 95 °C and 60 s at 60 °C. Baseline and threshold were automatically calculated and only adjusted manually when necessary.

## Results

The experimentally verified specificity of the screening PCRs which is needed for correct interpretation of the screening results in a given sample is shown in Table 4. The specificity of all screening methods included in the module was verified experimentally against a large set of reference materials (Table 1). In most cases the expected presence or absence of an element in the respective reference materials was confirmed (Table 4). Some element methods were not able to detect the related element as a result of sequence differences (indicated with an ”X” in Table 4) in the reference material tested. Most reactions that were expected to be negative were tested in practice as well. All element methods were able to detect the 25 copies of GMO sensitivity controls.

In some reference materials low level contaminations with other GMO events were detected that explained unexpected elements detected at high Cq values (ranging from 34 to 40). For example, in MON1445 reference material Cry1Ab/Ac and Cry1Ac elements were detected and confirmed by detection of traces of MON531 cotton. In MON531 and MON15985 cotton P-FMV, T-E9 CP4-epsps, and ctp2/CP4-epsps were detected and confirmed by detection of traces of MON1445 event. In the AOCS 1011-A and AOCS 0304-A, MON88302 DNA GT73 canola was detected, as well as traces of Rf3, Ms8 and the cotton event 3006-210-23.

**Fig. 2:**
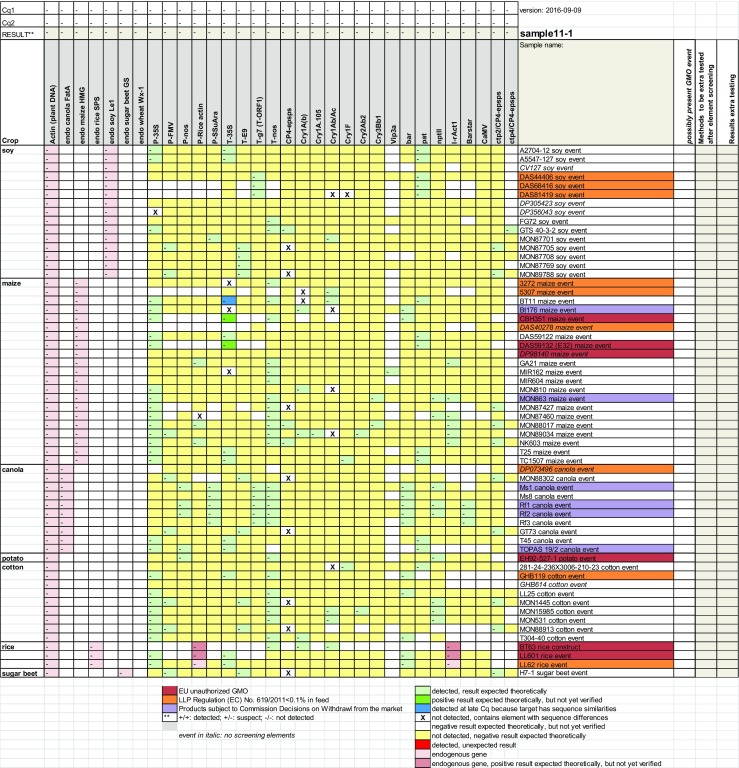
Overview of the analysis sheet (Online Resource 1). Authorised GMO events have a *white background*. GMOs that fall under the Regulation (EC) No. 619/2011 for low level presence (0.1%) in feed have an *orange background*. GMOs for which the authorisation has expired have a *purple background* and EU unauthorised GMOs have got a *red background*. The Cq values of the screening results are copied to the *two upper lanes* (Cq1 and Cq2). Then the results (*D* detected, *ND* not detected, *S* suspect) are automatically copied to the *third line* and to the *green cells* in the sheet. The boxes of screening results that are expected to be positive have a *light green background*, the results that are expected to be negative have a *light yellow background*

**Fig. 3:**
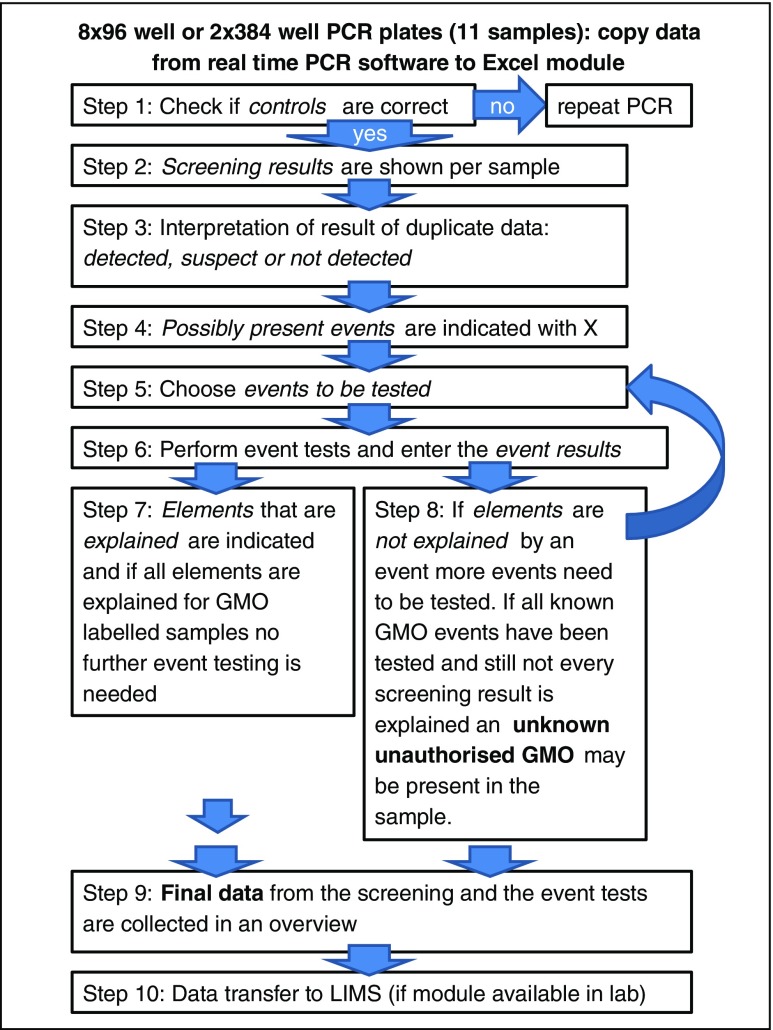
Extended screening strategy Excel module explained in ten steps (for more details see explanation tab in Online Resource 1)

The Microsoft Excel 2010 module is available as Electronic Supplementary Material (ESM) including the detailed procedure for use of the module to analyse laboratory results. A screen shot of an empty result sheet for one sample can be seen in Fig. 2. The authorisation status of the events is shown with explanatory colouring. On the basis of the detected elements the possible GMO events to be tested are indicated with an “X” in the lane “possibly present GMO event” (Fig. 2). In the next lane the results of the event testing can be entered (D, detected; ND, not detected). When this is completed the elements or constructs that are explained by the events detected will get a green background. In this way one can assess whether more GMO event tests need to be carried out to explain the elements and constructs detected in the sample. For GMO labelled samples no further tests are needed when all elements and constructs are explained (all screening elements and constructs have a green background). For non-GMO labelled samples all events that are indicated with an “X” in the lane “possibly present GMO event” should be tested. All detected events need to be quantified using event-specific methods to check if they comply with the 0.9% labelling threshold. A more general overview to show the different steps in the analysis module is given in Fig. 3. A detailed description of the Excel module is given in Online Resource 1.

## Discussion

In this article a setup is presented to efficiently test series of samples for GMOs. Series of 11 samples can be tested in a cost-effective way based on 32 screening methods. The screening tests are pipetted using a QIAgility robot in eight 96-well plates. On the basis of the combination of the detected and undetected endogenous genes, elements and constructs, a limited number of GMO events can be present in the sample. Especially in samples containing multiple GMOs this approach can reduce the amount of event-specific methods needed after the screening. When all elements are explained no further events need to be tested for GMO labelled samples. This means that probably not all authorised GMO events will be tested for. For instance, if a sample contains MON89034 maize and MON810 maize, the following elements will be positive: P35S, P-FMV, T-nos, cry1A(b), Cry1A.105, cry2Ab2 and I-rActin1. After confirming the MON89034 event, which contains all of these elements, all elements have been explained and MON810 maize (P-35S and cry1Ab) will not be tested for and MON810 will thus not be identified. Although it is theoretically feasible that the combination of identified elements and related events may mask the presence of one or more unauthorised GMO events, it is considered that the chance is low, and it will entail much work to further assess the sample in this case, as there is no obvious starting point to look for unauthorised GMOs. If a detected element cannot be explained by any known event this is an indication of an unknown unauthorised GMO in the sample. This possibility of obtaining indications of unknown unauthorised GMOs is an advantage in terms of enforcement strategies compared with a screening strategy employing only the minimal amount of elements needed to screen for all known GMO events.

The samples are tested for the presence of maize, soy, canola, wheat, potato, sugar beet and several elements and constructs. As a control for the DNA quality the actin gene, which should be positive in all plant materials, is also included. The actin gene can also serve as a positive control for more exotic samples for which no endogenous gene is available in the laboratory. On the basis of the practical experience that GTS-40-3-2 is detected in most samples, the Roundup Ready construct method was added to the standard screening. The specificity of the screening methods was verified in practice against a set of 59 GMO reference materials. This practical verification was deemed necessary because elements with the same name do not always have the same DNA sequence in different GMOs. Although the specificity can also be predicted in silico on the basis of the sequences of the primers, probe and the element, it is not always certain what the specificity will be in the actual PCR reaction in a specific reference material. Moreover, DNA sequence data are not yet available for all elements in all GMO events.

Most element methods showed the expected specificity outcome in the CRMs. In several cases GMO elements that were expected to be positive on the presence of an element with the same name in that particular GMO (http://www.euginius.eu/) are not detected in practice. This does not hamper the GMO screening as long as the correct specificity information is used for the interpretation of the screening results and as long as enough other screening elements are available for a given GMO. In most cases where elements are not picked up because of sequence differences, several other screening elements are still at hand for screening. For example the rice actin promoter present in MON87460 maize is not detected, but there are four other screening elements left that can be detected in MON87460 maize. Seven GMO events (in italics and marked with an asterisk in Table 4) contain no GMO elements that are part of the screening strategy as applied and need to be tested with their event-specific method. As an alternative a P-35S method that does detect DP356043 soy is also available [12].

The screening strategy contains several elements derived from donor organisms (e.g. *npt*II which is present in e.g. *Escherichia coli* and T-g7 from *Agrobacterium tumefaciens*). If such an element is found to be positive it is necessary to take into account the possibility that the donor organism is the cause of the positive signal and not a GMO. This problem can also occur with elements derived from other plants (e.g. T-E9 from pea, P-Rice actin and I-*rAct*1 from rice). The screening strategy cannot detect stacked events (and neither can other screening strategies), although similar Cq values for individual events can be an indication of the presence of a stack.

Some of the specificity results did not agree with information published earlier [20]. The P-35S [26] was not detected by us in DP98140 maize but was reported as detected by Block et al. [20] The DP98140 maize contains no P-35S according to the EUginius database. T-35S [17] was found to be positive in T45 canola and DAS59122 maize and reported as unexpectedly positive by Block et al. According to the EUginius database both GMOs contain a T-35S so a positive signal can be expected in T45 canola and DAS59122 maize. For Bt11 maize T-35S signals with comparable Cq as the other elements were found (marked with + * in Table 4). As these signals are too low to suspect contamination with other reference these may be caused by sequence similarities between Bt11 maize and the T-35S primers and probe. The table from Block et al. does not contain T-35S information on Bt11 maize.

The screening results can be evaluated in a semiautomatic way using a Microsoft Excel module which is available as ESM (Online Resource 1). This module can also be used to plan the subsequent event-specific testing. Depending on the sample labelling all possible GMO events indicated by the Excel module need to be tested for and quantified (non-GMO labelling) or only the event tests that explain all detected elements need to be carried out (for GMO labelled samples). The screening results per sample can be seen graphically in relation to the detected GMO events and their authorisation status. In case not all elements are explained extra events may need to be tested, or the (unknown) GMO needs to be identified using other techniques like next generation sequencing [8]. All analysis results are automatically summarised in an overall table by the Excel module, in a format that will allow the laboratory to send the data in one action to a laboratory information management system that is compatible with Excel. In this way this GMO analysis protocol and data analysis module can help enforcement as well as other laboratories to analyse series of samples in a highly informative and time- and cost-effective way.

## Electronic supplementary material

Below is the link to the electronic supplementary material.

ESM 1 (PDF 492 kb)

ESM 2 (XLSX 1942 kb)
