# Supplementary material for: Semiautomated TaqMan PCR screening of GMO labelled samples for (unauthorised) GMOs
Source: Anal Bioanal Chem. 2017 Apr 17;409(15):3877–89. doi: 10.1007/s00216-017-0333-7 (PMC5427157; doi:10.1007/s00216-017-0333-7)
Supplement: Supplementary file 1 — (PDF 492 kb) [file 216_2017_333_MOESM1_ESM.pdf]

Analytical and Bioanalytical Chemistry

Electronic Supplementary Material

Semiautomated TaqMan PCR screening of GMO labelled samples for (unauthorised) GMOs

Ingrid M.J. Scholtens, Bonnie Molenaar, Richard A. van Hoof, Stephanie Zaaijer, Theo W. Prins, Esther J. Kok

Additional file (216_2017_333_MOESM2_ESM.xlsx) available under "Supplementary Material"
